# Dexamethasone Palmitate Ameliorates Macrophages-Rich Graft-versus-Host Disease by Inhibiting Macrophage Functions

**DOI:** 10.1371/journal.pone.0096252

**Published:** 2014-05-07

**Authors:** Satoshi Nishiwaki, Takayuki Nakayama, Makoto Murata, Tetsuya Nishida, Seitaro Terakura, Shigeki Saito, Tomonori Kato, Hiroki Mizuno, Nobuhiko Imahashi, Aika Seto, Yukiyasu Ozawa, Koichi Miyamura, Masafumi Ito, Kyosuke Takeshita, Hidefumi Kato, Shinya Toyokuni, Keisuke Nagao, Ryuzo Ueda, Tomoki Naoe

**Affiliations:** 1 Department of Hematology and Oncology, Nagoya University Graduate School of Medicine, Nagoya, Aichi, Japan; 2 Japan Society for the Promotion of Science, Japanese Red Cross Nagoya First Hospital, Nagoya, Aichi, Japan; 3 Department of Hematology, Japanese Red Cross Nagoya First Hospital, Nagoya, Aichi, Japan; 4 Department of Transfusion Medicine, Aichi Medical University, Nagakute, Aichi, Japan; 5 Department of Pathology, Japanese Red Cross Nagoya First Hospital, Nagoya, Aichi, Japan; 6 Department of Cardiology, Nagoya University Graduate School of Medicine, Nagoya, Aichi, Japan; 7 Department of Pathology and Biological Responses, Nagoya University Graduate School of Medicine, Nagoya, Aichi, Japan; 8 Department of Dermatology, Keio University School of Medicine, Shinjyuku-ku, Tokyo, Japan; 9 Department of Tumor Immunology, Aichi Medical University, Nagakute, Aichi, Japan; New York University, United States of America

## Abstract

Macrophage infiltration of skin GVHD lesions correlates directly with disease severity, but the mechanisms underlying this relationship remain unclear and GVHD with many macrophages is a therapeutic challenge. Here, we characterize the macrophages involved in GVHD and report that dexamethasone palmitate (DP), a liposteroid, can ameliorate such GVHD by inhibiting macrophage functions. We found that host-derived macrophages could exacerbate GVHD in a mouse model through expression of higher levels of pro-inflammatory TNF-α and IFN-γ, and lower levels of anti-inflammatory IL-10 than resident macrophages in mice without GVHD. DP significantly decreased the viability and migration capacity of primary mouse macrophages compared to conventional dexamethasone *in vitro*. DP treatment on day 7 and day 14 decreased macrophage number, and attenuated GVHD score and subsequent mortality in a murine model. This is the first study to provide evidence that therapy for GVHD should be changed on the basis of infiltrating cell type.

## Introduction

Macrophages are recruited by chemokines including CCL2 to the inflammatory site and, primarily, play an indispensable role in both innate and acquired immunity [1]. Macrophage phenotypes and functions can vary with different external stimuli, and macrophages are divided into two major classifications: classically activated, i.e. inflammatory, and alternatively activated, i.e. anti-inflammatory macrophages [1]. Persistence of activated macrophages can occasionally be harmful to the host [2], [3].

Graft-versus-host (GVHD) is often a prominent complication after allogeneic stem cell transplantation (allo-SCT) and can be fatal despite aggressive interventions including corticosteroids [4]. It has been reported that GVHD can be divided into 3 subtypes based on the number of macrophages and T lymphocytes infiltrated in the skin, and that GVHD with many CD163^+^ macrophages was refractory with poor prognosis and a therapeutic challenge [5]. However, those macrophages were CD163 positive, a member of the scavenger receptor cysteine-rich superfamily, which was one of anti-inflammatory macrophage markers [6]. Thus, the pathogenesis between macrophage infiltration and refractory GVHD is currently unclear. These facts prompted us to characterize the phenotypes of macrophages related to refractory GVHD.

Corticosteroids inhibit functions of inflammatory cells via glucocorticoid receptors in the cytoplasm [7]. Therefore, efficient delivery of corticosteroids into the cytoplasm could enhance their therapeutic effect. It is known that a dexamethasone palmitate emulsion (DP) is readily taken up by macrophages via phagocytosis and is strongly retained in the cytoplasm [8]. Here, we report that the macrophages increased in fatal GVHD are inflammatory and that DP treatment efficiently attenuated such GVHD by inhibiting macrophage functions.

## Materials and Methods

### 1. Mice

Male 6- to 8-week-old male C57BL/6J mice and female BALB/c mice were purchased from Chubu Kagaku Shizai (Nagoya, Japan). The animal GVHD experiments using spontaneous death as an endpoint were approved by the Institutional Ethics Committee for Laboratory Animal Research, Nagoya University School of Medicine (protocol 24298), and were performed according to the guidelines of the institute. Animals were maintained at constant ambient temperature (22±1°C) under a 12-h light/dark cycle (lights on between 9:00 and 21:00), with food and water available ad libitum.

### 2. Cells and reagents

A murine macrophage cell line, RAW264.7, was purchased from American Type Culture Collection (Manassas, VA, USA). Primary peritoneal macrophages and skin macrophages were obtained from the peritoneal lavages of C57BL/6J mice and from the ears of mice after BMT, respectively, as described elsewhere with slight modification [9], [10]. Briefly, peritoneal lavages were collected 3 days after intraperitoneal injection of 1 mL of 2% thioglycolate (Kanto Chemical Co., Inc., Tokyo, Japan) and macrophages were positively selected from the lavages by AutoMACS system with anti CD11b immunomagnetic microbeads (Miltenyi Biotec, Bergisch Gladbach, Germany). To prepare single cell suspensions from dermis, the ears were split into dorsal and ventral halves with removal of subcutaneous tissues such as cartilage and the dermal sheets were incubated in RPMI containing 2% Liberase (Liberase TL Research Grade, Roche Applied Science) for 2 hours at 37°C. After digestion, residual tissue was minced in RPMI and disaggregated by using a tissue homogenizer (Medimachine; Becton Dickinson, San Jose, CA). Dermal macrophages were positively selected from the suspension cells by AutoMACS as described above. FACS analysis using a monoclonal antibody (mAb) (F4/80: BM8, BioLegend, San Diego, CA, USA) showed that the purity of isolated macrophages were >90%. Primary T cells were positively isolated from splenocytes of BALB/c mice by using anti CD90.2 immunomagnetic microbeads (Miltenyi Biotec). T cell-depleted (TCD) donor bone marrow (BM) cells was obtained from BALB/c mice by negative selection by using CD90.2 microbeads.

Dexamethasone sodium phosphate (DSP) and Dexamethasone palmitate emulsion (DP) were from MSD K.K. (Tokyo, Japan) and Mitsubishi Tanabe Pharma (Tokyo, Japan), respectively.

### 3. Induction and assessment of GVHD

A fatal murine GVHD model was established by allogeneic BM transplantation. Lethally irradiated C57/BL6 recipient mice (5 Gy×2; days -2 and -1) were co-transplanted with TCD-BM (5×10^6^) and T lymphocytes (1×10^7^) from BALB/c donor mice via tail vein without anesthesia. DP or DSP (10 mg/kg as dexamethasone) were administrated intravenously into the mice on day 7 and 14 after transplantation (control: n = 9, DSP: n = 9, DP: n = 10). The conditions and survival of animals after BMT were monitored daily with all efforts to alleviate pain and suffering, and the degree of GVHD was evaluated clinically (3 times/week) for 28 days (until day 42) after the last administration of DP or DSP (day 14) because our preliminary experiments showed that GVHD-related complications were neither worsen nor cause of death after 28 days of DSP treatment. The reasons why we set spontaneous death as an endpoint are as follows. GVHD also possesses an antitumor effect; so-called graft-versus-leukemia (GVL), and ‘mild’ GVHD confers a survival benefit [11] [12]. Thus, physicians try to modulate the GVL-GVHD balance by immune-suppressants such as steroid and cyclosporine A. However, GVHD, once became refractory to conventional therapies, could cause high mortalities [13]. To determine whether DP can improve overall survival outcomes or not in a GVHD mouse model brings a lot of useful information to physicians. The animals survived by DP treatment were humanely euthanized by overexposure to carbon dioxide after day 42. Mice treated with no steroid or with DSP had all died of GVHD before day 42. The detailed clinical GVHD scoring system by using 5 parameters is as follows: weight loss, posture, activity, fur texture, and skin integrity (maximum index = 10) [14]. Acute GVHD was also assessed in a blind fashion by detailed histopathologic analysis in hematoxylin and eosin-stained tissue sections (the skin from the interscapular region, ears and descending colon). Skin sections were scored on the basis of the following criteria: epidermis (0, normal; 1, foci of interface damage in <20% of section with occasional necrotic keratinocytes; 2, widespread interface damage in >20% of section); dermis (0, normal; 1, slightly altered with mild increased collagen density; 2, marked increased collagen density); inflammation (0, none; 1, focal infiltrates; 2, widespread infiltrates); subctaneous fat (0, normal; 1, reduced number of normal adipocytes; 2, serous fat atrophy); and follicles (0, normal number of hair follicles, ∼5 per linear millimeter;1, between 1 and 5 follicles per linear millimeter; 2, <1 follicle per linear millimeter) [15]. Seven parameters were scored for gut (crypt regeneration, crypt epithelial cell apoptosis, crypt loss, surface colonocyte vacuolization, surface colonocyte attenuation, lamina propria inflammatory cell infiltrate, and mucosal ulceration). The scoring system for each parameter denoted 0 as normal; 0.5 as focal and rare; 1 as focal and mild; 2 as diffuse and mild; 3 as diffuse and moderate; and 4 as diffuse and severe, as previously described [16].

To assess the direct effect of inflammatory macrophages on GVHD, 1×10^6^ thioglycolate-stimulated peritoneal macrophages from C57BL/6J mice were subcutaneously injected in interscapular region on day 5. All mice were humanely euthanized by overexposure to carbon dioxide on day 7 and GVHD score was pathologically evaluated as described above.

### 4. Analysis of donor-cell chimerism

Donor-cell chimerism of macrophages in the skin after BMT was analyzed by FACS using anti-MHC haplotype antibodies. An anti-H-2Kb mAb (AF6-88.5) and an anti- H-2Kd (SF1-1.1) recognized cells from C57/BL6 recipient mice and cells from BALB/c donor mice, respectively. Both mAbs were obtained from PharMingen (San Diego, CA).

### 5. RNA preparation and real-time PCR analysis

Total RNA was extracted from the skin and gut of mice using TRIzol (Invitrogen Carlsbad, CA, USA). The mRNA levels of CCL2 in the skin and gut, and those of TNF-α, IFN-γ and IL-10 in skin macrophages were evaluated using quantitative RT-PCR. Primer pairs (TNF-α: Mm00443258_m1, IFN-γ: Mm01168134_m1, CCL-2: Mm00441242_m1, IL-10: Mm00439614_m1, Arginase-1: Mm00475988_m1, Eukaryotic 18s rRNA: 4333760T) were from Applied Biosystems (Foster City, CA, USA). Obtained data were normalized to internal 18 s rRNA expression and were analyzed using the 2^−ΔΔ^C_T_ Method [17].

### 6. *In vitro* assay for the effects of DP on macrophages and lymphocytes

The viability of RAW264.7 after DSP or DP treatment was assessed using a colorimetric assay as described elsewhere [18]. Briefly, 10 µl of TetraColor-One (Seikagaku Co., Tokyo, Japan) was added to each well of a 96-well plate, where RAW264.7 cells (10,000 cells/well) were pretreated with various concentrations of DSP or DP (48 hours, 37°C), and the mixture was incubated for an additional 4 hours. Absorbance at 450 nm was monitored. The viability of splenic T lymphocytes after exposure to DSP or DP was assessed by trypan blue exclusion. Briefly, cells were washed twice with PBS, suspended in culture medium (RPMI containing 10% FBS), plated (1.0×10^5^ cells/well in 0.2 mL culture medium) in three independent determinations with DSP or DP (25 nM) onto 96-well plates, and incubated for 48 hours. Viable cells were determined as Trypan blue- negative cells. The percent viability was calculated as follows: (viability in DSP or DP group/viability in control group) ×100 (%).

CCR2 expression on the surface of macrophages after DSP or DP treatment was analyzed by FACS as described previously [19]. Briefly, thioglycolate-recruited peritoneal macrophages were pretreated with 25 µM (as dexamethasone) of DSP or DP for 3 hours, and then, were exposed to LPS (O55:B5, 100 ng/mL, List Biological Laboratories, Inc. Campbell, CA) for 18 hours. After washings with PBS, the cells were incubated for 30 minutes on ice with a rabbit anti-mouse CCR2 polyclonal antibody (pAb) (1: 25 dilution, E68, Novus Biologicals, Littleton, CO) in the presence of an anti-mouse CD16/32 mAb (BioLegend, San Diego, CA) to reduce non-specific binding of a primary antibody to Fc receptor. The cells were washed twice with PBS and were incubated with a fluorescein isothiocyanate -conjugated donkey anti-rabbit IgG pAb (1: 25 dilution, BioLegend, San Diego, CA) as a secondary antibody for 30 minutes. CCR2 expression was assessed from 1.0×10^4^ viable cells using a FACSAria flow cytometer (BD Biosciences, San Jose, CA), and the data were analyzed using FlowJo software (TreeStar, San Carlos, CA). Background fluorescence was assessed through staining with the isotype-matched antibody.

To assess the inhibitory effect of DP and DSP on the CCL2-CCR2 axis, transwell migration assays were performed as described elsewhere [18]. Briefly, RAW264.7 cells were pretreated with DSP or DP (25 µM) for 3 hours and then, were serum-starved in DMEM with 1% FBS and 100 ng/ml LPS overnight. After washing, the cells were seeded (2×10^5^ cells in DMEM with 1% FBS per well) onto the upper chamber of a cell culture insert with a pore size of 8 µm (BD Biosciences, San Jose, CA). Recombinant murine CCL2 (final concentration, 20 ng/ml; PeproTech, Rocky Hill, NJ) was added to the lower chamber, to which cells were allowed to migrate for 4 hours. The membranes were fixed with 4% paraformaldehyde and were stained with Giemsa. For quantitative analysis, four fields were randomly selected, and migrated cells were counted under a light microscope.

### 7. Immunohistochemistry

Immunostaining of murine skin and gut specimens was carried out on sections from paraffin-embedded tissues fixed in 10% neutral-buffered formalin solution (Sigma-Aldrich, St. Louis, MO) using streptavidin-biotinylated HRP detection (Beckeman Coulter, Brea, CA) as previously described with slight modification [20]. For antigen retrieval, sections (3.5 µm thickness) on silane-coated slides were heated in a microwave oven for 45 minutes at 98°C in immunosaver (1∶200 dilution, Nisshin EM Corp. Tokyo, Japan). After blocking nonspecific binding with normal rabbit serum (1∶75 dilution; Dako Inc. Via Real (Carpinteria, CA), sections were incubated with an anti F4/80 mAb (CI:A3-1, Novus, Littleton, CO) (1∶100) or an isotype-matched mAb for 15 minutes using intermittent microwave irradiation [21], [22]. Sections were then incubated with biotin-labeled rabbit anti-mouse IgG pAb (1∶300 dilution; Dako Inc.) and 3,3′-diaminobenzidine (DAB; Vector Laboratories Inc. Burlingame, CA) was used as chromogen. Finally they were counterstained with hematoxylin.

### 8. Statistical Analysis

Statistical significance of group differences was evaluated using Student's *t*-test between two groups and ANOVA followed by bonferroni test for multiple comparisons using STATA software (StataCorp, Lakeway, TX). Kaplan-Meier product-limit estimates were performed to determine survival, while the different subgroups were compared for significance using the log-rank test.

## Results

### 1. Murine GVHD model mimics human severe GVHD

**Figure 1:**
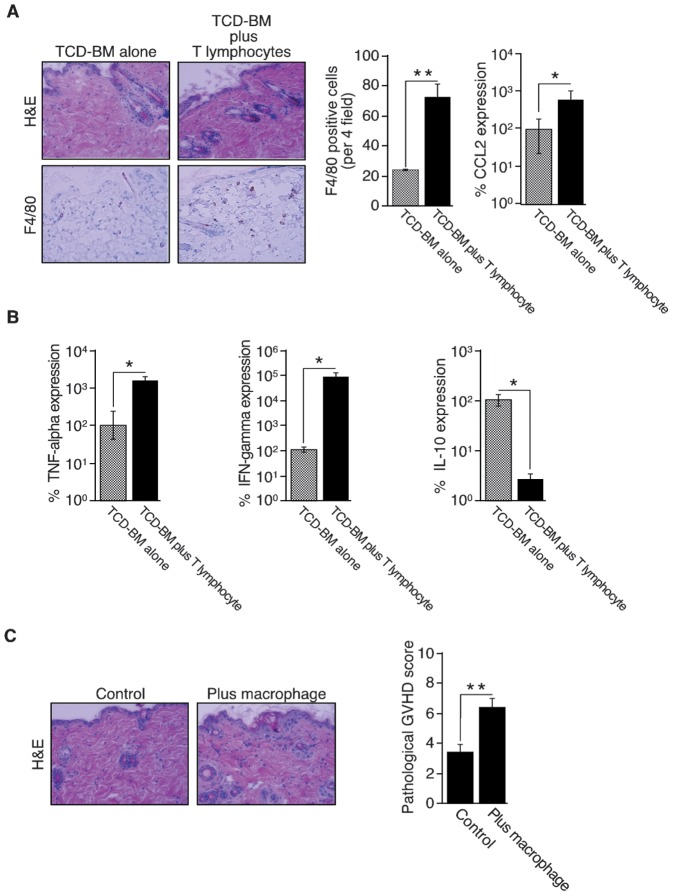
Characterization of macrophage-involvement in GVHD. **A:** Skin specimens from mice in which T-cell depleted bone marrow (TCD-BM) cells and spleen-derived T lymphocytes were co-transplanted were histopathologically compared with those of mice transplanted with TCD-BM alone. Skin specimens (7 days after transplantation) were stained with H&E (upper left panels) and macrophages were detected with the anti-mouse F4/80 monoclonal antibody (lower left panels). Original magnification, ×400. Representative images of two independent experiments are shown. For quantitative analysis of macrophage numbers, 4 microscopic fields were randomly selected from each of three mice and the total number of F4/80^+^ cells was counted. The mean number of F4/80^+^ cells per 4 fields ± SD is shown for each group (middle panel). Original magnification, ×200. The results are representative of two independent experiments. Statistical significance: **P<0.01. CCL2 mRNA expression was evaluated using quantitative real-time RT-PCR. Total RNA was extracted from the skin (3 specimens per group) and was subjected to RT-PCR using specific primer pairs. Each reaction was performed in duplicate sets. The obtained data were normalized to internal eukaryotic 18 s rRNA expression, were analyzed using the 2^−ΔΔ^C_T_ Method and are expressed as percent expression, where expression of the control is designated as 100%. The results reflect the mean ± SD of three independent determinations. The results are representative of two independent experiments. Statistical significance: *P<0.05. **B:** Macrophages in the skin were characterized by measurement of TNF-α, IFN-γ and IL-10 mRNA expression. Skin dermis was incubated in RPMI containing 2% Liberase for 2 h at 37°C. After digestion, residual tissue was minced and mechanically disaggregated. After separation of dermal macrophages by using the magnetic isolation system, total RNA was extracted and mRNA levels of TNF-α, IFN-γ and IL-10 were quantified by RT-PCR using specific primer pairs (3 specimens per group). Each reaction was performed in duplicate sets. The obtained data were normalized to internal eukaryotic 18 s rRNA expression, were analyzed using the 2^−ΔΔ^C_T_ Method and are expressed as percent expression, where expression of the control is designated as 100%. The results reflect the mean ± SD of three independent determinations. Statistical significance: *P<0.05. The results are representative of two independent experiments. **C:** Direct effects of inflammatory macrophages on GVHD were assessed by injecting thioglycolate-stimulated macrophages (1×10^6^ cells per mouse) or PBS (three mice per group) subcutaneously into the interscapular region of GVHD mice 5 days after BMT. The animals were killed 7 days after BMT. Skin specimens of the injected sites stained with H&E were photographed (left panel) and skin GVHD was pathologically scored based on five parameters (epidermal damage, alteration of dermis, degree of inflammation, alteration of subcutaneous fat and number of follicles). The results reflect the mean ± SD of three independent determinations. Statistical significance: **P<0.01. The results are representative of two independent experiments.

In a BMT model, mice received TCD-BM (5×10^6^) and T lymphocytes (1×10^7^) or TCD-BM (5×10^6^) alone. Cotransplantation of TCD-BM and T lymphocytes resulted weight loss, poor activity, damaged fur texture and all of the mice died on day 12 even though all of the mice received TCD-BM alone were active and survived. Pathological analysis of the skin showed necrotic keratinocytes, increased collagen density, infiltration of inflammatory cells and serous fat atrophy in mice with TCD-BM and T lymphocytes, but minimal damage in mice with TCD-BM alone (Figure 1A, left panel). The gut was similarly severely damaged in mice with TCD-BM and T lymphocytes (data not shown). A higher number of macrophages infiltrated the skin of mice had received TCD-BM and T lymphocytes compared to the skin of mice had received TCD-BM alone (Figure 1A, middle panel). These results clearly suggest that this fatal mouse GVHD model mimics human severe GVHD with many macrophages [5].

To analyze the mechanism of macrophage infiltration in the skin, we focused on the role of CCL2-CCR2 axis since CCL2 is a potent inducer of macrophage recruitment and activation [23]. Quantitative RT-PCR analysis showed that CCL2 expression in the skin of mice with TCD-BM and T lymphocytes was 10-times higher than that in the skin of mice with TCD-BM alone (Figure 1A, right panel).

### 2. Characterization of macrophages increased in GVHD

The phenotypes of dermal macrophages isolated on day 7 after BMT (the purity of macrophages >90%, not shown) were evaluated by quantitative RT-PCR analyses. Macrophages from GVHD mice showed that skin macrophages from GVHD mice expressed much higher levels of TNF-α and IFN-γ, and a significantly lower level of IL-10 than those of sham mice (no GVHD) (Figure 1B), suggesting that the macrophages involved in GVHD possess inflammatory properties [1]. To assess the direct effect of inflammatory macrophages on GVHD, 1×10^6^ thioglycolate-stimulated peritoneal macrophages from C57BL/6J mice were subcutaneously injected in the interscapular region on day 5 and evaluated on day 7. Skin pathological score of the injected site was significantly higher among mice injected macrophages than PBS-injected control mice (Figure 1C). Donor-cell chimerism analyzed by FACS showed that >90% of dermal macrophages possessed the recipient phenotype (data not shown). These data indicated that recipient monocytes recruited to the skin GVHD site acquired inflammatory phenotypes and deteriorated GVHD subsequently.

### 3. Effects of DP on macrophage functions in vitro

**Figure 2:**
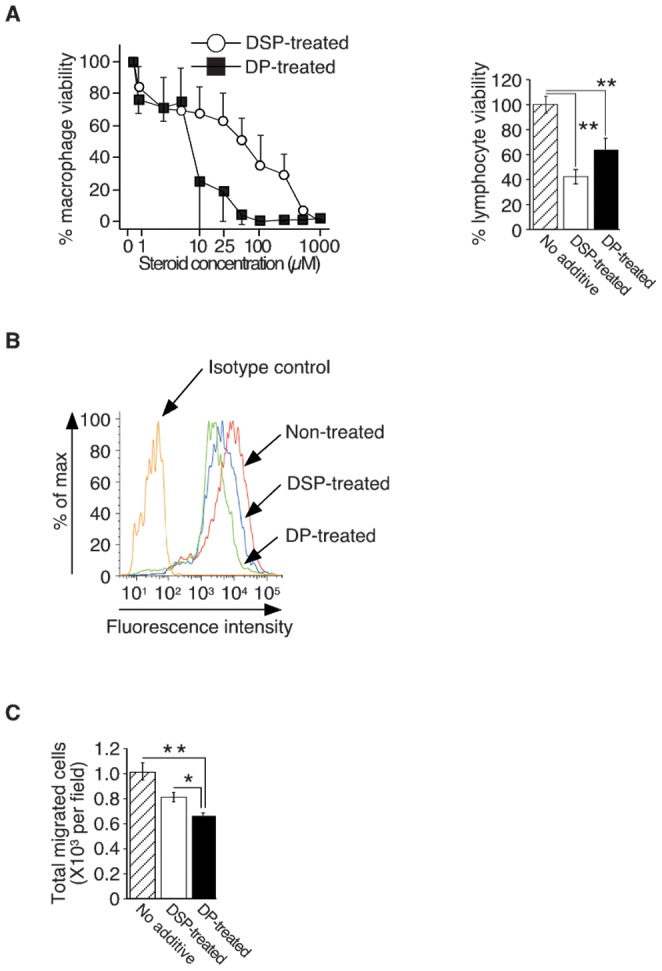
Effect of dexamethasone palmitate on macrophages *in vitro*. **A:** The viability of mouse macrophage-like RAW264.7 cells after dexamethasone sodium phosphate (DSP) or dexamethasone palmitate (DP) treatment (48 hours) was evaluated by using a colorimetric assay (left panel). The percentage viability was calculated as follows: (O.D value in the presence of each concentration of steroid/O.D value without steroid) ×100. The results reflect the mean ± SD of three independent determinations (representative experiment of three performed). The viability of splenic T lymphocytes after exposure to DSP or DP (25 nM each, 48 hours) was assessed by trypan blue exclusion (right panel). Viable cells were determined as Trypan blue- negative cells. The percent viability was calculated as follows: (viability in DSP or DP group/viability in control group) ×100 (%). The results reflect the mean ± SD of three independent determinations (representative experiment of three performed). **B:** CCR2 expression on the surface of mouse primary peritoneal macrophages after DSP or DP treatment was evaluated by FACS. The results are representative of three independent experiments (left panel). **C:** The migration of peritoneal macrophages towards CCL2 after DSP or DP treatment was analyzed using transwell assays. For quantitative analysis, four fields were randomly selected, and migrated cells were counted under a light microscope (×200). The results reflect the mean ± SD of four independent determinations. Representative results of three independent experiments are shown (right panel). Statistical significance: *P<0.05 and **P<0.01.

Based on these results, we hypothesized that GVHD with many macrophages would be ameliorated by inhibiting macrophage functions. We therefore compared DP with conventional DSP on macrophage functions. Both DSP and DP inhibited proliferation of RAW 264.7 cells in a dose dependent manner. However, DP possessed a significantly higher ability than DSP (Figure 2A left panel). DP decreased the viability of RAW 264.7 cells by 75% at a concentration of 10 µM, which is 25-fold lower than the concentration at which DSP similarly worked (by 71% at 250 µM) (Figure 2A left panel). Interestingly, the toxic effect of DP on splenic T lymphocytes is rather weak toxic than DSP, when tested at 25 µM as dexamethasone (Figure 2A right panel). DP also significantly decreased CCR2 expression on the surface of primary peritoneal macrophages (Figure 2B) and RAW 264.7 cells (data not shown), and subsequently decreased migration of primary macrophages towards CCL2 (Figure 2C) compared to DSP. This decreased number of macrophage migration could not be attributed to decreased number of the input cells, as 3 hour-treatment with DSP or DP at 25 µM minimally affected on macrophage viabilities (data not shown). These results clearly suggest that DP attenuates macrophage functions more efficiently than DSP.

### 4. The effect of DP on murine fatal GVHD

**Figure 3:**
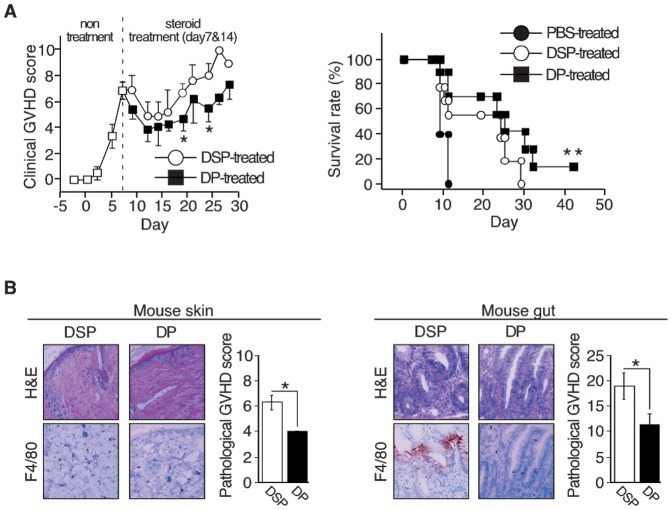
Effect of dexamethasone palmitate on macrophages in mice with fatal GVHD. **A:** Clinical assessment of GVHD after DP or DSP treatment. Mice were treated with DP or DSP (10 mg/kg/each day) on day 7 and day14 after co-transplantation of TCD-BM and spleen-derived T lymphocytes (control: n = 9, DSP: n = 9, DP: n = 10). Clinical GVHD was assessed 3 times a week, using a scoring system consisting of 5 clinical parameters: weight loss, posture, activity, fur texture, and skin integrity (maximum index = 10, left panel). Statistical significance: *P<0.05 (days 19 and 24). Mortalities were counted daily for up to 42 days after transplantation (right panel). Statistical significance: **P<0.01 (PBS-treated versus DP-treated). **B:** Pathological assessment of GVHD after DP or DSP treatment. Skin (left panels) and gut (right panels) specimens were stained with H&E (top panels) and skin macrophages were detected with the anti-mouse F4/80 monoclonal antibody (bottom panels). Original magnification, ×400. Representative images of the skin of three mice are shown. Skin and gut GVHD were scored based on five parameters (epidermal damage, alteration of dermis, degree of inflammation, alteration of subcutaneous fat and number of follicles) and seven parameters (crypt regeneration, crypt epithelial cell apoptosis, crypt loss, surface colonocyte vacuolization, surface colonocyte attenuation, lamina propria inflammatory cell infiltrate and mucosal ulceration), respectively, and are presented as histograms. Statistical significance: *P<0.05.

We next investigated whether DP could affect the fatal murine GVHD with many macrophages. DP or DSP was administered 10 mg/kg as dexamethasone on day 7 and day14 (control: n = 9, DSP: n = 9, DP: n = 10). DP significantly lowered the clinical GVHD score compared to DSP. The difference became apparent 5 days after second administration (Figure 3A, left panel). Subsequently DP could rescue about 20% of these mice, whereas mice treated with no steroid or with DSP had all died by days 12 and 30, respectively (Figure 3A, right panel). The effect of DP was also confirmed by pathological analyses, where tissue damage and pathological GVHD scores in the skin (Figure 3B, left panel) and gut (Figure 3B, right panel) were significantly improved in mice treated with DP. The number of F4/80^+^ macrophages was also lower in mice treated with DP. Since DP had a weaker effect on lymphocytes than DSP (Figure 2A, right panel), the combined facts suggest that DP can attenuate GVHD with many macrophages by inhibiting inflammatory macrophages.

## Discussion

Macrophage infiltration in the skin of patients with GVHD is a maker of poor prognosis [5]. Here, we identify macrophages in the GVHD sites are inflammatory and an exacerbator of GVHD, and provide evidence that such GVHD can be effectively treated by DP, not conventional DSP in a mouse model.

Inflammatory cells such as macrophages and mast cells have been proved to be durable to even high dose chemotherapy and irradiation [24], [25]. Accordingly, donor-cell chimerism analysis showed that >90% of dermal macrophages possessed the recipient phenotype (not shown).

Macrophages are divided into two major classifications: classically activated, i.e. inflammatory, and alternatively activated, i.e. anti-inflammatory macrophages [26]. Since persistence of macrophage activation can be harmful to the host, phenotypic switch from inflammatory macrophages to anti-inflammatory macrophages can be occurred via various stimuli [27]. We revealed by RT-PCR that macrophages in the skin of a murine GVHD model possessed inflammatory properties (Figure 1B) although the macrophages in patients with GVHD expressed CD163 [5], a marker of the alternatively activated macrophages [26]. Recently, compelling studies revealed that CD163^+^ macrophages could be unrestrained proinflammatory macrophage population with an incomplete switch to anti-inflammatory macrophages under certain circumstances such as iron-overloading condition [28]. An elevated level of ferritin, a marker of tissue iron overload, closely correlates with increased risk of acute GVHD, higher mortality and lower overall survival [29]. These evidences and results suggest that CD163^+^ macrophages in patients with GVHD can be inflammatory and exacerbate GVHD similarly with the mouse GVHD model.

CCL2-CCR2 signaling is known to play a major role in recruitment of monocytes/macrophages [23]. Inflammatory mediators released from activated macrophages not only induce tissue damage but also recruit and activate macrophages [1]. DP treatment in a mouse GVHD model decreased the number of macrophages in the skin and gut, and attenuated GVHD without severe complications compared to DSP treatment (Figure 3), suggesting that DP inhibited the positive feedback loop between macrophages and inflammation more efficiently than by DSP.

Not a few attempts to prevent severe acute GVHD in animal models by inhibiting macrophage function as an antigen-presenting cell or modulating macrophage phenotype have been reported and some were successful [30]–[35]. However, severe adverse effects such as infections occurred occasionally [31] since inflammatory macrophages play important roles in both innate and acquired immune response. Minimal risk of infection can give DP treatment an advantage over those pretreatment.

Our small preclinical study showed that DP treatment in patients with macrophage-rich GVHD (3 day, 5 mg/day) ameliorated GVHD with efficient reduction of skin macrophages. Severe adverse effects such as infections were not observed during and after DP treatment (not published).

Again, these observations provide further evidence that macrophages directly exacerbate GVHD and that DP treatment against such macrophages improves the outcome of refractory GVHD.
